# Smart Composite Hydrogels with pH-Responsiveness and Electrical Conductivity for Flexible Sensors and Logic Gates

**DOI:** 10.3390/polym11101564

**Published:** 2019-09-26

**Authors:** Tong Wang, Xuan Zhang, Zichao Wang, Xiuzhong Zhu, Jie Liu, Xin Min, Tao Cao, Xiaodong Fan

**Affiliations:** 1 The Key Laboratory of Space Applied Physics and Chemistry, Ministry of Education and Shaanxi Key Laboratory of Macromolecular Science and Technology, School of Science, Northwestern Polytechnical University, Xi’an 710072, China; 17795837806@163.com (T.W.); wc19910102@126.com (Z.W.); zhuxiuzhong321@163.com (X.Z.); liujie509_1982@126.com (J.L.); 15991672082@163.com (X.M.); caotao@mail.nwpu.edu.cn (T.C.); 2 School of Light Industry and Engineering, Qi Lu University of Technology (Shandong Academy of Sciences), Jinan 250353, China

**Keywords:** stimuli-responsive conductive hydrogels, pH-responsive, composite hydrogels, flexible sensors, logic gates

## Abstract

Stimuli-responsive conductive hydrogels have a wide range of applications due to their intelligent sensing of external environmental changes, which are important for smart switches, soft robotics, and flexible sensors. However, designing stimuli-responsive conductive hydrogels with logical operation, such as smart switches, remains a challenge. In this study, we synthesized pH-responsive conductive hydrogels, based on the copolymer network of acrylic acid and hydroxyethyl acrylate doped with graphene oxide. Using the good flexibility and conductivity of these hydrogels, we prepared a flexible sensor that can realize the intelligent analysis of human body motion signals. Moreover, the pH-responsive conductive hydrogels were integrated with temperature-responsive conductive hydrogels to develop logic gates with sensing, analysis, and driving functions, which realized the intellectualization of conductive hydrogels.

## 1. Introduction

Conductive hydrogels have mechanical flexibility and controllable electronic properties, and are one of the hottest materials for electronic sensors [1,2], flexible skins [3,4], supercapacitors [5,6,7], and nerve electrodes [8]. With the development of smart devices, more and more studies are focusing on smart materials [9,10,11,12,13]. The intellectualization of conductive hydrogels endues conductive hydrogels with stimuli-responsive functions, so that they can intelligently respond to external stimuli and produce changes in volume [14]. However, due to the complexity of environmental changes and the difficulty in constructing smart devices, using stimuli-responsive conductive hydrogels to construct complex smart devices remains a huge challenge in current scientific research.

Recently, a number of studies have been conducted on stimuli-responsive conductive hydrogels, which can be used to realize the intellectualization of flexible sensors [14,15], drug release [16], and logical controllers [17]. Poly(*N*-isopropylacrylamide) (PNIPAM) was used to construct temperature-sensitive conductive hydrogels [17,18,19,20], which showed a good response to temperature and high electrical conductivity for making temperature-controlled switches [21,22]. However, for logic switches with intelligent control, one type of stimuli was not enough, due to the complexity of environmental changes [23]. At the same time, few studies reported pH-responsive conductive hydrogels [24,25,26,27,28] and did not focus on developing intelligent control devices [28,29]. Therefore, by mimicking the integrated circuit, the combination of two or more types of stimuli-responsive materials may achieve the fabrication of the device [23].

Hydrogel logic control devices have the advantage of convenient manipulation and reusability, with wide applications in the fields of flexible electronics and regenerative medicine. Kim et al. [30] used hydrogels as polyelectrolytes to prepare a pH-controlled organic electrochemical transistor, and applied it to electrochemical logic circuits to develop NOT, NOR, and NAND logic gates. Huang et al. [10] prepared novel ion-conducting supramolecular hydrogel with reversible photoconductivity, controlling the resistance of the circuit by switching the light source and applying it to the logic circuit to selectively control the on/off state of the circuit by light. Hu et al. [31] used semi-interpenetrating hydrogel film to achieve logical control of electroactive probes, providing a novel and convenient model for constructing multi-signal switchable bioelectrocatalysis. Deforest et al. [32] used a modular design to prepare hydrogel logic gates for drug delivery by combining different stimuli responsiveness. However, the aforementioned hydrogels are complicated in terms of preparation. For practical production, how to use the simple synthesis method to prepare hydrogels for constructing logic control devices is a problem worth considering.

In order to solve this problem, we needed to prepare a pH-responsive conductive hydrogel. In general, polyacrylic acid hydrogels have good pH-responsiveness, which is commonly used as the drug controlled release material [33,34]. However, acrylic hydrogels are very brittle, which is a big problem in building smart devices [35]. Therefore, many studies have been reported on improving the mechanical properties of polyacrylic acid hydrogels to prepare smart devices, such as flexible sensors [36,37]. Herein, in the process of polymerization of acrylic acid and hydroxyethyl acrylate monomers into hydrogels, we added graphene oxide to a prehydrogel solution to prepare pH-responsive conductive hydrogels, which have high conductivity. At the same time, the addition of hydroxyethyl acrylate monomer effectively improved the mechanical properties of the acrylic hydrogel, and the conductive hydrogels had good flexibility. A flexible sensor with high sensitivity was fabricated to realize rapid monitoring and the feedback of human activity. Furthermore, we combined pH-responsive conductive hydrogels with temperature-sensitive conductive hydrogels to develop logic gates, such as YES, OR, and AND gates with intelligent control functions, which considerably enriched the application field of intelligent conductive hydrogels.

## 2. Experimental Section

### 2.1. Materials

Graphene oxide (GO) was synthesized according to the previous method [26]. *N*-isopropylacrylamide (NIPAM) was purchased from TCI. Acrylic acid (AA) was purchased from Tianjin Fuchen Chemical Reagents Factory. *N*,*N*-methylenebisacrylamide (BIS) and *N*,*N*,*N*′,*N*′-tetramethylethylenediamine (TMEDA) were purchased from J&K. 2-Hydroxyethylmethacrylate (HEMA) was purchased from Aladdin. Aniline (99.5%) was purchased from Aladdin and distilled under reduced pressure. Phytic acid (50% in water) was purchased from Aladdin. Ammonium persulfate (APS; 98%) was purchased from Guangdong Guanghua Sci-Tech Co., Ltd. Ethylene Glycol dimethacrylate (EGDMA; 98%) was purchased from Innochem (Beijing, China), and citric acid (99%) was purchased from Energy Chemical (Shanghai, China). Disodium hydrogen phosphate dodecahydrate was purchased from Guangdong Guanghua Sci-Tech Co., Ltd (Guangdong, China). The 184 Silicone Elastomer was purchased from Dow Corning (Midland, Michigan, US) for preparing polydimethylsiloxane (PDMS).

### 2.2. Preparation of pH-Responsive Conductive Hydrogels

**Figure 1:**
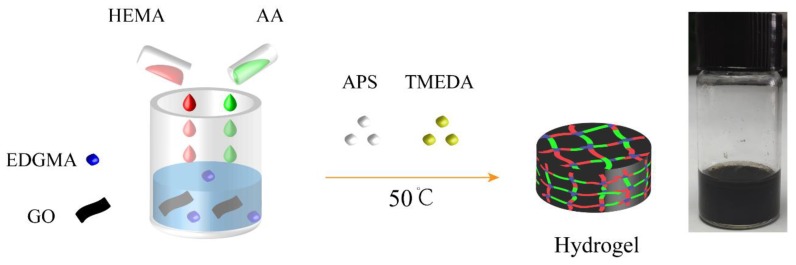
Schematic of Poly(acrylic acid-*co*-2-hydroxyethyl acrylate)/graphene oxide (P(AA-HEMA)/GO) hydrogels: the red segments are Poly(2-hydroxyethyl acrylate) (PHEMA), the green segments are Poly(acrylic acid)(PAA), and the blue segments are chemical crosslinking points.

The preparation process of pH-responsive conductive hydrogels is illustrated in Figure 1. In a typical process, 2 mL of deionized water, 0.41 g of AA, and 2.93 g of HEMA were mixed with 20 mg of GO in a vial. After vigorously stirring for 2 h, ultrasonication was carried out for 1 h to uniformly disperse GO in the solution. Then, 33.3 mg of EDGMA, 0.3 mL (5 wt %) of APS solution, and 0.2 mL of TMEDA were separately added to the solution. The vial was purged with nitrogen gas for 30 min, and the gelation process was conducted in an oven at 50 °C for 3 h. To remove the unreacted monomers, the hydrogel was taken out and immersed in water for 24 h.

### 2.3. Preparation of Flexible Sensor

A flexible sensor was manufactured to detect the change in resistance of the hydrogel, and to monitor large-scale human motion. We used the P(AA-HEMA)/GO conductive hydrogel as a conductor between the two aluminum foils, and encapsulated the sensor using an elastic tape to protect the hydrogel and prevent moisture evaporation. For monitoring movement of the human body, the sensor was attached to the finger and the wrist. The aluminum foils were connected to the Keithley electrometer (Keithley 2450) at an AC voltage of 2 V, and the output resistance was measured. Different actions (such as compression, stretching, and distortion) were performed, and the electrometer was used to detect the electrical change of the hydrogel, indicating the high sensitivity of the conductive gel.

### 2.4. Assembling of Logic Gate Device 

First, we used the PDMS prepolymer liquid to make the device. The mold was printed using a 3D printer and consisted of two differently sized cuboids. We then placed two pieces of aluminum foil on both sides of the mold in a plastic petri dish and poured in the degassed PDMS prepolymer solution. After the petri dish was placed in an oven at 50 °C for 12 h, the cured PDMS was taken out from the petri dish. In this manner, we obtained PDMS with the expected structure. We then separately cut the temperature-sensitive conductive hydrogels (Supporting Information in Section 1) and pH-sensitive conductive hydrogels, and placed the two hydrogels in the groove of the PDMS. We inserted two pieces of aluminum foil into the circuit with a bulb and power.

### 2.5. Measurements

Scanning electron microscopy (SEM, VEGA3-LMH, TESCAN Corp., Brno, Czech Republic) was employed to observe the surface morphologies of the freeze-dried hydrogels. The Fourier transform infrared (FTIR) spectra of the freeze-dried hydrogels was obtained using a PerkinElmer Spectrum Two FTIR spectrometer in the range of 450–4000 cm^−1^. The conductivity of the hydrogels was measured using a SX1944 digital four-probe tester. Human activity and hydrogel electrical changes were tested using an electrometer (Keithley 2450). For simultaneous detection, the hydrogel was attached to the corresponding skin portion and the aluminum foil was attached to the Keithley 2450. The mold that was used to construct the logic gate device was printed using a 3D printer (Form 2, Formlabs). The pH of the configured pH buffer solution was measured using a pH meter (PHS-2F).

## 3. Results and Discussion

### 3.1. Chemical Structures of pH-Responsive Conductive Hydrogels

**Figure 2:**
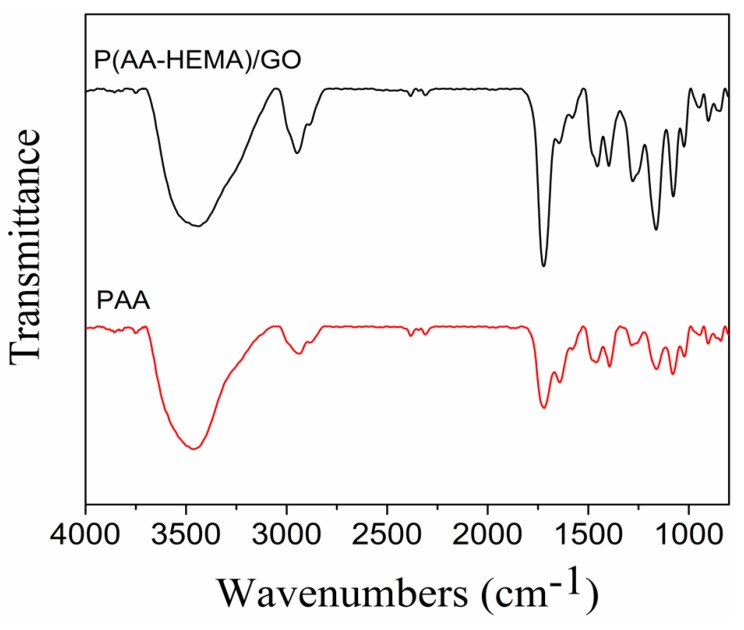
FTIR spectra of P(AA-HEMA)/GO hydrogels (**black**) and PAA hydrogels (**red**).

The chemical structure of P(AA-HEMA)/GO hydrogels was identified using FTIR spectroscopy. Figure 2 shows the FTIR spectra of PAA hydrogels and P(AA-HEMA)/GO hydrogels. The results show that the infrared absorption line of PAA exhibited a stretching vibration absorption peak of C = O in the carboxyl group at 1724 cm^−1^. Note that a broad absorption peak appeared around 3440 cm^−1^, which can be attributed to the stretching vibration of the hydroxyl group of AA and the hydroxyl group of HEMA. 

**Figure 3:**
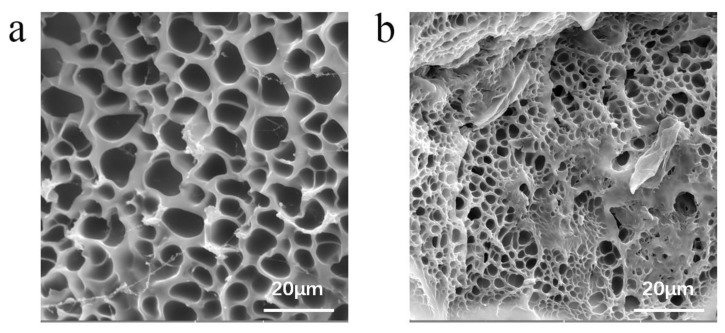
SEM images of (**a**) PAA hydrogels and (**b**) P(AA-HEMA)/GO hydrogels.

We obtained the microstructure of the hydrogels from freeze-dried hydrogel samples using scanning electron microscopy (SEM). The microstructure is crucial for pH-responsive hydrogels, which require water to rapidly go into or out of the whole gel. As shown in Figure 3a, the hydrogels presented a homogenous and porous microstructure. This structure indicated the presence of interconnected channels in the hydrogels, which considerably improved the response rate because of the fast transport of water through the gel.

As shown in Figure 3b, there were many lamellar structures in the porous structure of the P(AA-HEMA)/GO hydrogel under the same magnification, which indicated the successful preparation of the PAA and GO composite materials. Moreover, by observation, we determined that the pore structure of the P(AA-HEMA)/GO hydrogels was smaller than the pure PAA hydrogels because of the following reasons [26]: the GO surface was hydrophilic and the sheet was compatible with the matrix, forming a dense polymer network structure; and the hydrogels contained the GO sheet, which prevented the growth of pores during the freeze-drying process. Figures S1 and S2 show the chemical structures of temperature-responsive conductive hydrogels.

### 3.2. pH-Responsiveness and Conductivity of P(AA-HEMA)/GO Hydrogels

**Figure 4:**
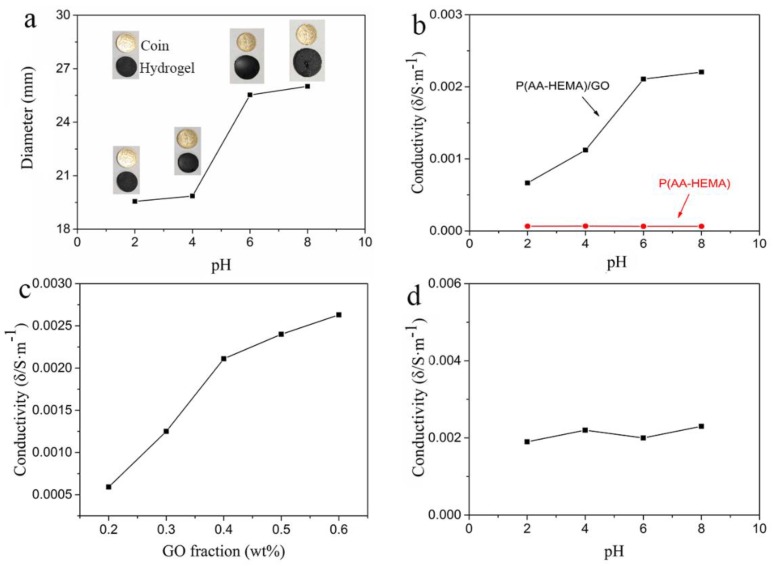
(**a**) The diameter of the hydrogels varies with an increase in pH from 2 to 8 (0.15 M Na_2_HPO_4_ and citric acid buffer solution). The yellow circles represent the coin and the black circles represent the hydrogel; (**b**) pH-dependent conducting properties for P(AA-HEMA)/GO and P(AA-HEMA) hydrogels; (**c**) electrical conductivity of the hydrogels with different GO ratios; (**d**) pH-dependent conducting properties for PHEMA/GO hydrogels without AA.

The P(AA-HEMA)/GO hydrogel contained a number of –COOH groups that showed an outstanding pH-responsiveness. Because the degree of dissociation of –COOH varied under different pH conditions, the hydrogel composites showed different degrees of swelling. As shown in Figure 4a, the hydrogel remained in a contracted state at pH < 4. Even if the buffer concentration reached 4, the pH inside the hydrogel was still less than the pKa (4.3) of AA. The dissociation degree of the gel network was low, and electrostatic repulsion hardly contributed to the gel′s swelling. Therefore, when at pH < 4, the gel slightly swelled with the increase in pH. When at pH > 4, the dissociation degree of the gel network rapidly increased with ion exchange inside and outside of the hydrogel. Moreover, the electrostatic repulsion was considerably enhanced, and the diameter of the hydrogel was drastically increased [38,39]. 

As shown in Figure 4b, when the pH increased, the conductivity of the hydrogel increased. This may have been caused by the change in the swelling states and microstructures of the hydrogels [19]. As the pH increased, electrostatic repulsion occurred, due to the continuous ionization of –COOH in the hydrogel, and the hydrogel volume increased. The water in the hydrogel increased, and the density of the polymer backbone decreased, which facilitated the transfer of electrons on graphene oxide. In comparison, we can see that the conductivity of P(AA-HEMA) was very small. As the pH changed, the conductivity remained essentially the same, which showed that the main carrier was electron from GO. We explain the change in diameter and conductivity with the temperature of PINPAM hydrogels in Figure S3. 

As shown in Figure 4c, we judged that the percolation threshold was about 0.35 wt %, and the GO concentration in the hydrogel was close to the threshold for GO percolation. As shown in Figure 4d, we prepared hydrogels without acrylic acid under the same conditions. We found that in changing the pH, the conductivity of the hydrogels remained almost unchanged, indicating that the –COOH in acrylic acid had little effect on the conductivity of the hydrogels. At the same time, the –COOH in GO had very little effect on the conductivity of the hydrogels. Therefore, the main carrier was electron from GO.

### 3.3. Wearable Devices for Monitoring Human Motions

**Figure 5:**
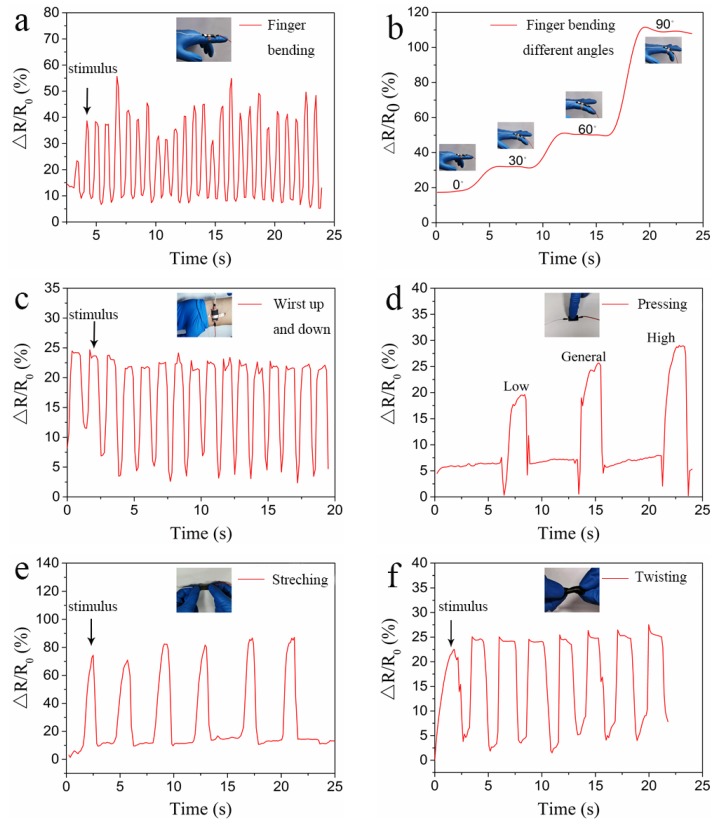
Wearable flexible sensors based on P(AA-HEMA)/GO hydrogels. (**a**) the sensor for monitoring the motion of the fingers, the (related original resistance of the sensor) *R*_0_ was 1155 Ω; (**b**) the hydrogel sensors adhered to the gloves with the finger bending at different angles, the *R*_0_ was 1115 Ω; (**c**) the sensor for monitoring the motion of the wrist, the *R*_0_ was 1092 Ω; (**d**) pressing the hydrogel sensor to detect its sensitivity, the *R*_0_ was 6300 Ω; (**e**) stretching, the *R*_0_ was 3968 Ω; (**f**) twisting, the *R*_0_ was 5134 Ω. The size of the hydrogels in (**a–c**) were different from the size of the hydrogels in (**d–f**).

The pH-responsive conductive hydrogel was assembled as a flexible wearable smart device for detecting human activity. The shape of the gel was rectangular parallelepiped. The length was 4.5 cm, the width was 1.5 cm, and the thickness was 1.5 mm. When the flexible sensor was used for human body detection, the hydrogel was constantly stretched and linked with real-time measuring equipment by electrical wires. The sensing response was calculated as relative resistance change, as follows:

S (%) = (*R* − *R*_0_)/*R*_0_ × 100% = Δ*R*/*R*_0_ × 100% (1)

where *R*_0_ and *R* are the related original resistance of the sensor and the resistance after stretching, respectively. As shown in Figure 5, the relative resistance change of the conductive hydrogel gradually increased when the hydrogel was stretched. This can be explained by the stretching of the conductive hydrogel, which narrowed the porous microstructure, and damaged the 3D network framework of GO for electron transport, giving rise to the growth of resistance [40].

As shown in Figure 5a–c, the flexion of the finger was detected using a flexible sensor to show the hydrogel’s rapid response. Moreover, when the fingers were bent at different angles, the hydrogel attached to the fingers was stretched to different lengths, and thus the resistance was different, thereby outputting signals of different heights. Similarly, for wrist activity, when the hydrogel was mounted on the wrist, mechanical motions of the wrist were monitored by these output curves. 

As shown in Figure 5d, the sensor distinguished the degree of pressure during compression. Different pressures corresponded to different resistance changes and output different waveforms. Since the hydrogels were the copolymers of AA and HEMA, the hydrogels had flexibility and stretchability. Besides pressure, the specific waveform was output when we stretched (Figure 5e) and twisted (Figure 5f) the sensor. Notably, the resistance-time curves of the three mechanical stimuli showed high signal-to-noise ratios, which indicated high sensitivity and reliability of the flexible sensor. As shown in Figure S4, the hydrogels exhibited different geometries under different motions. Figure S5 indicates the stability of the sensor.

### 3.4. The Preparation of Logic Gates Based on pH-Responsive and Thermo-Responsive Conductive Hydrogels 

**Figure 6:**
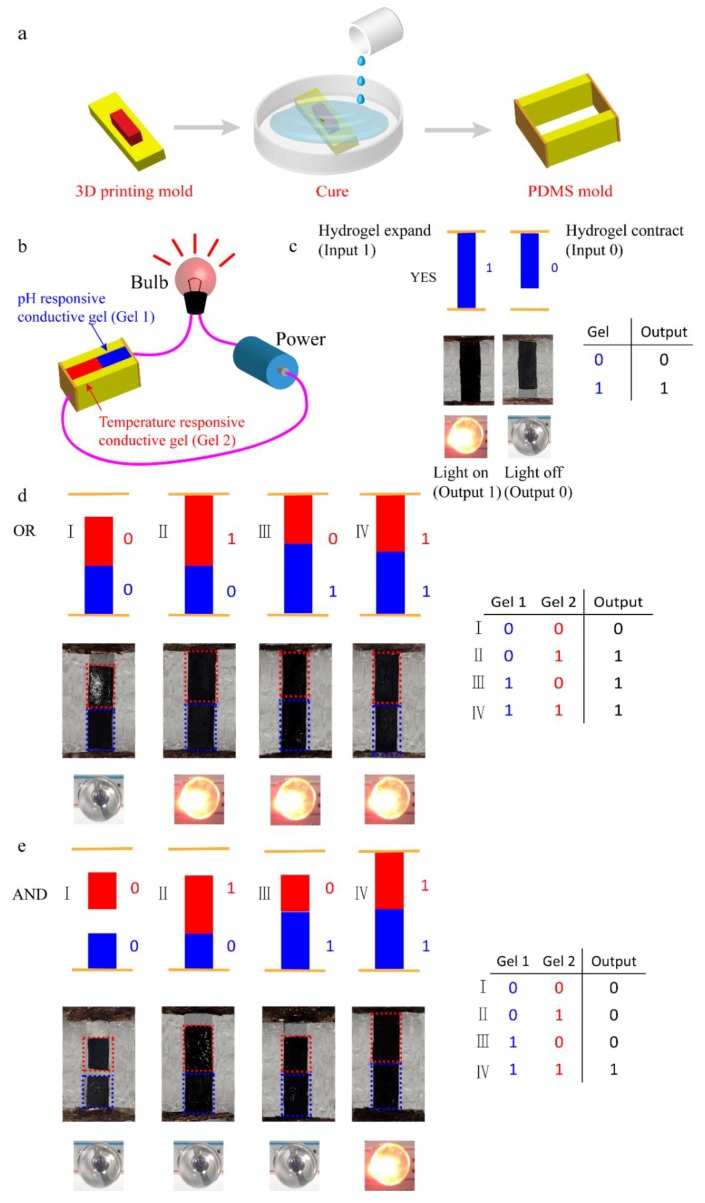
The design of logic gates. Schemes illustrating (**a**) preparation of molds using polydimethylsiloxane (PDMS); (**b**) using power, a bulb, PDMS molds, and stimuli-responsive conductive hydrogels to form logic gates. The red rectangles represent temperature-responsive conductive hydrogels and the blue rectangles represent pH-responsive conductive hydrogels; (**c**) thedifferent states of the YES gates and the truth tables; (**d**) the different states of the AND gates and the truth tables; (**e**) the different states of the OR gates and the truth tables.

As shown in Figure 6a, the shape of the printing mold was copied by the PDMS. As illustrated in the inset of Figure 6b, we built the circuit using power, a bulb, PDMS molds, and stimuli-responsive conductive hydrogels. As shown in Figure 6c, we specified a state input of 0 when the hydrogel shrunk, and a state input of 1 when the hydrogel expanded. When the lamp was unlit, we specified the output to be 0; however, when the lamp was lit, we specified the output to be 1. First, we used pH-responsive conductive hydrogels to make the YES logic gate and to prove our method. At pH 2, when the hydrogel was exposed to a solution, it shrunk (input 0) and the bulb went out (output 0); however, at pH 8, the hydrogel expanded (input 1) and the bulb illuminated (output 1); this process was reversible. Similarly, we developed the YES logic gate using temperature-responsive conductive hydrogels (Figure S6, Supporting Information).

Using the specific design of the logic system, we built more complex logic gates. Figure 6d,e show the methods of making OR and AND logic gates using stimuli-responsive conductive hydrogels. The devices included two stimuli-responsive hydrogels that respond to different stimuli. For the OR gate, the two hydrogels were adjacent to each other. Whenever any of the hydrogels expanded under the influence of the stimulus, the hydrogel expanded, the circuit turned on, and the bulb was illuminated (output 1). When both hydrogels were stimulated at the same time, they expanded and the circuit turned on (output 1). For the AND gate, we maintained a certain distance between the two hydrogels. When only one hydrogel expanded, it only expanded into the space and connected to another hydrogel, but did not turn on the circuit, and the bulb did not illuminate (output 0). Therefore, the circuit would only turn on when both hydrogels swelled, after which the bulb would illuminate (output 1). As shown in Figures S7 and S8 (Supporting Information), we provide the size of the logic gates and the expansion ratios of the two responsive hydrogels. By combining the expansion ratios and the dimensional changes of the hydrogels, the preparation of the logic gate device can be better understood. We experimentally verified the expected execution of these two logic gates for all input states. The demonstrations show that we used different stimuli to respond to conductive hydrogels and controlled the position of the hydrogels, such that we developed different types of chemical logic gates.

We constructed the logic gates using the integration of two stimulus responsive materials. A logic gate is a smart device that intelligently senses changes in the external environment, thereby driving its own volume change and achieving intelligent control. Connecting the logic gate to the circuit and constructing the intelligent switch as the control light bulb is one of the important applications of the logic gate. In addition, intelligent controlled release and molecular probes for drugs can be achieved by using logic gates.

## 4. Conclusions

In summary, we introduced a simple method of preparing pH-responsive conductive hydrogels. PAA was introduced as the pH-responsive component, GO was introduced as the conductive component, and PHEMA was introduced to increase flexibility. In the buffer solution of pH 2 to pH 8, the conductive hydrogel gradually increased in diameter and conductivity due to the gradual increase of the degree of ionization of the carboxyl group and the ion concentration in the hydrogel. A highly sensitive flexible sensor was fabricated using a flexible pH-responsive conductive hydrogel for monitoring human motions. At the same time, the two kinds of stimuli-responsive hydrogels were combined to sense the environment, regulate the volume change of the hydrogel, and realize intelligent control, thereby expanding the application range of the conductive hydrogel and making the chemical materials intelligent.

## Supplementary Materials

The following are available online at https://www.mdpi.com/2073-4360/11/10/1564/s1, Figure S1: FT-IR spectra of PNIPAM/PANI hydrogels (black) and PNIPAM hydrogels(red)., Figure S2: SEM images of PNIPAM hydrogels (a) and PNIPAM/PANI hydrogels (b). Figure S3: (a) The diameter of the hydrogels vary under increasing temperature from 2 to 50 °C. (b) The temperature-dependent conducting properties for PNIPAM/PANI hydrogels. Figure S4: The geometry for the electrodes by different motions. Figure S5: Real-time monitoring of relative resistance changes. Figure S6: Using temperature responsive conductive hydrogels to prepare YES gate. Figure S7: The size of the logic gates(The unit is cm). Figure S8: The swelling ratios of the hydrogels. (a) The pH-responsive conductive hydrogel. D_0_ represents the diameter of the hydrogel at pH 2, and ΔD represents the difference between the diameter of the other pH and D_0_. (b) The thermo-responsive conductive hydrogels. D_0_ represents the diameter of the hydrogel at 50 °C, and ΔD represents the difference between the diameter of the other temperature and D_0._
