# Supplementary material for: Smart Composite Hydrogels with pH-Responsiveness and Electrical Conductivity for Flexible Sensors and Logic Gates
Source: Polymers (Basel). 2019 Sep 26;11(10):1564. doi: 10.3390/polym11101564 (PMC6836247; doi:10.3390/polym11101564)
Supplement: Supplementary file 1 [file polymers-11-01564-s001.pdf]

SUPPORTING INFORMATION

Smart Composite Hydrogels With pH-Responsiveness and Electrical Conductivity for Flexible Sensors and Logic Gates

Tong Wang[1], Xuan Zhang[1]*, Zichao Wang[1], Xiuzhong Zhu[2], Jie Liu[1], Xin Min[1], Tao Cao[1] and Xiaodong Fan[1]*

1 The Key Laboratory of Space Applied Physics and Chemistry, Ministry of Education and Shaanxi Key Laboratory of Macromolecular Science and Technology, School of Science, Northwestern Polytechnical University, Xi'an, 710072, PR China

2 School of Light Industry and Engineering, Qi Lu University of Technology (Shandong Academy of Sciences), Jinan, 250353, P. R., China

* Corresponding author.E-mail addresses: zhangxuan@nwpu.edu.cn; xfand@126.com

1. Preparation of temperature-responsive conductive hydrogels.

The temperature-responsive conductive hydrogels were synthesized according to the previous method[1]. In a typical process, 3 mL deionized water were mixed with 0.6 g NIPAM monomer and 0.01 g N,N-methylenebisacrylamide (BIS) as crosslinker. Then 0.1 mL (5% wt) APS solution and 0.06 mL TEMED were added to the mixture and polymerization was carried out for 12 h at room temperature. The hydrogel was taken out and immersed in water for 24 h to remove the unreacted monomers. After cleaning, the hydrogel was immersed in 0.5 mol/L aniline solution to absorb the aniline monomer. After 12 h, the hydrogel was immersed in the solution of 6.85 g APS and 1.2 g phytic acid for 12 h. Finally, the obtained hybrid hydrogel was immersed in deionized water to remove unreacted residues.

2. The Chemical structures of temperature-responsive conductive hydrogels.

The chemical structures of PNIPAM/PANI and PNIPAM were analyzed by Fourier transform infrared spectroscopy (FTIR). Figure 1 shows the FTIR spectra of PNIPAM and PNIPAM/PANI. In the spectrum (red) of PNIPAM, peaks at 1558 cm^{-1} and 1645 cm^{-1} can be attributed to the N–H bending and C=O stretching, which are both characteristic peaks of PNIPAM. The infrared spectrum (black) is the infrared spectrum of PNIPAM / PANI hydrogel. All the characteristic peaks of PNIPAM can be found in the FTIR spectrum of PNIPAM / PANI. At the same time, there are two characteristic absorption peaks at 1562 cm^{-1} and 1508 cm^{-1} corresponding to the stretching vibration of the anthracene ring and the benzene ring, respectively. Therefore, formation of the PNIPAM and PANI composites was confirmed.

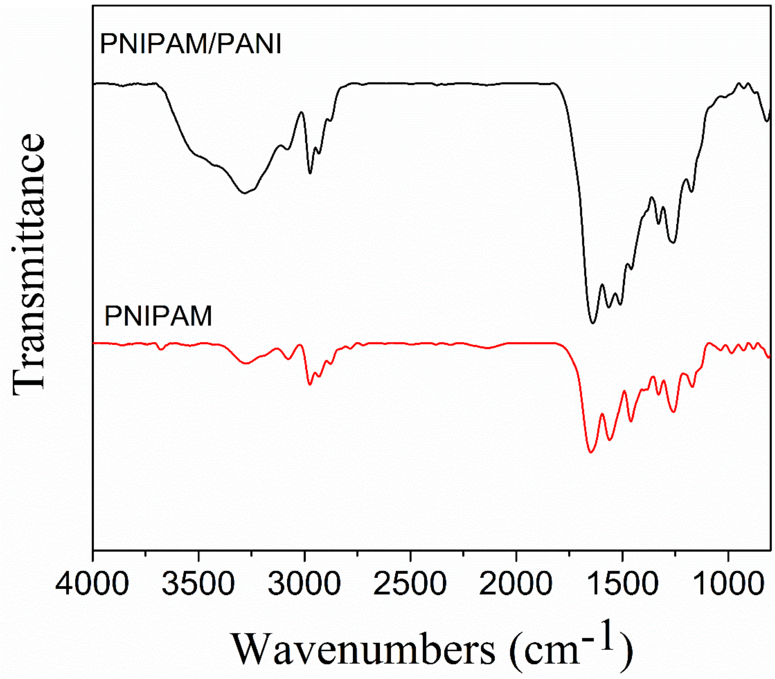

Figure S1. FT-IR spectra of PNIPAM/PANI hydrogels (black) and PNIPAM hydrogels (red).

The PNIPAM hydrogels have the porous structure that effectively absorbs aniline and oxidant into the hydrogel network for in situ polymerization. Macroscopically, the colorless PNIPAM hydrogel became a black PNIPAM/PANI hydrogel during this process. From Figure b, the pores of the PNIPAM/PANI hybrid hydrogel are smaller than those of the PNIPAM hydrogel, indicating that the network structure of the hydrogel is more dense, which further indicates the formation of an interpenetrating network structure in the system.

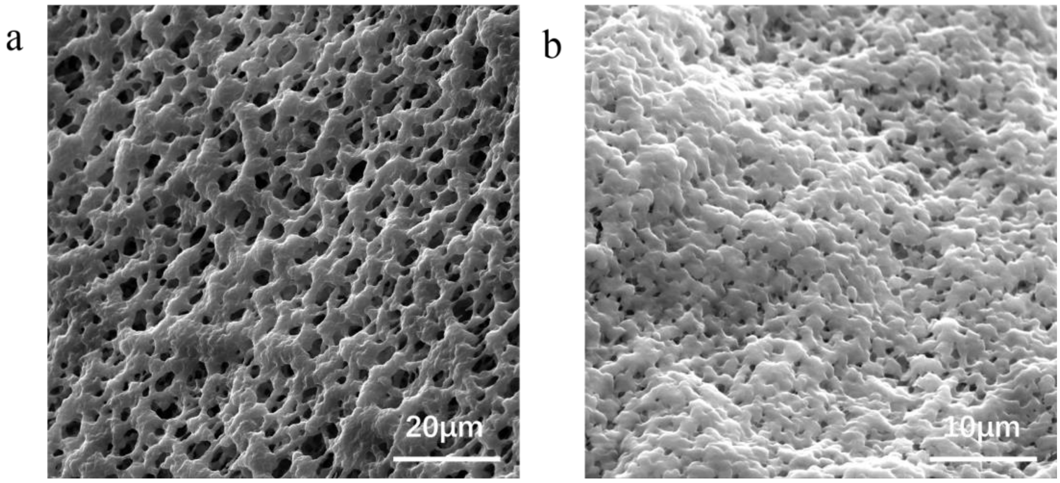

Figure S2. SEM images of PNIPAM hydrogels (a) and PNIPAM/PANI hydrogels (b).

3. The temperature-responsive and Conductivity of PNIPAAm/PANI hydrogels

When the temperature-sensitive conductive hydrogel is heated from 2 ° C to 50 ° C, the diameter of the hydrogel is reduced from 22 mm to 14 mm, where slope of the

change is greatest at 32 °C, which is critical solution temperature (LCST) (about 32 °C) of PNIPAM. At the same time, it was found that the conductivity of the hydrogel also varies with the temperature. This is because when the temperature changes, the volume of the hydrogel also changes due to the expansion or contraction. When the temperature increases, the hydrogel shrinks in volume, the water content decreases, and the polymer skeleton density increases, which hinders ion migration. Therefore, the conductivity of the hydrogel gradually decreases[2].

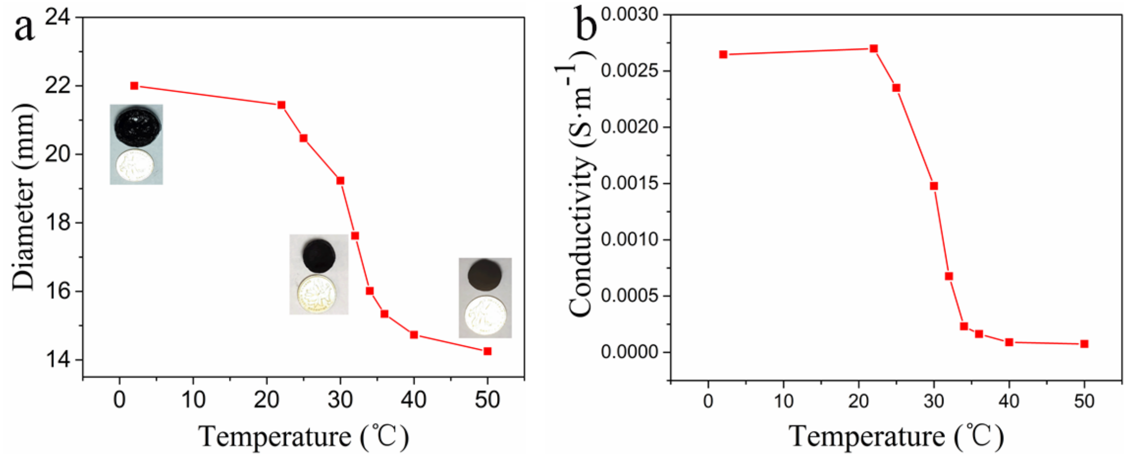

Figure S3. (a) The diameter of the hydrogels vary under increasing temperature from 2℃ to 50℃. (b) The temperature-dependent conducting properties for PNIPAM/PANI hydrogels.

4. The geometry for the hydrogels by different motions.

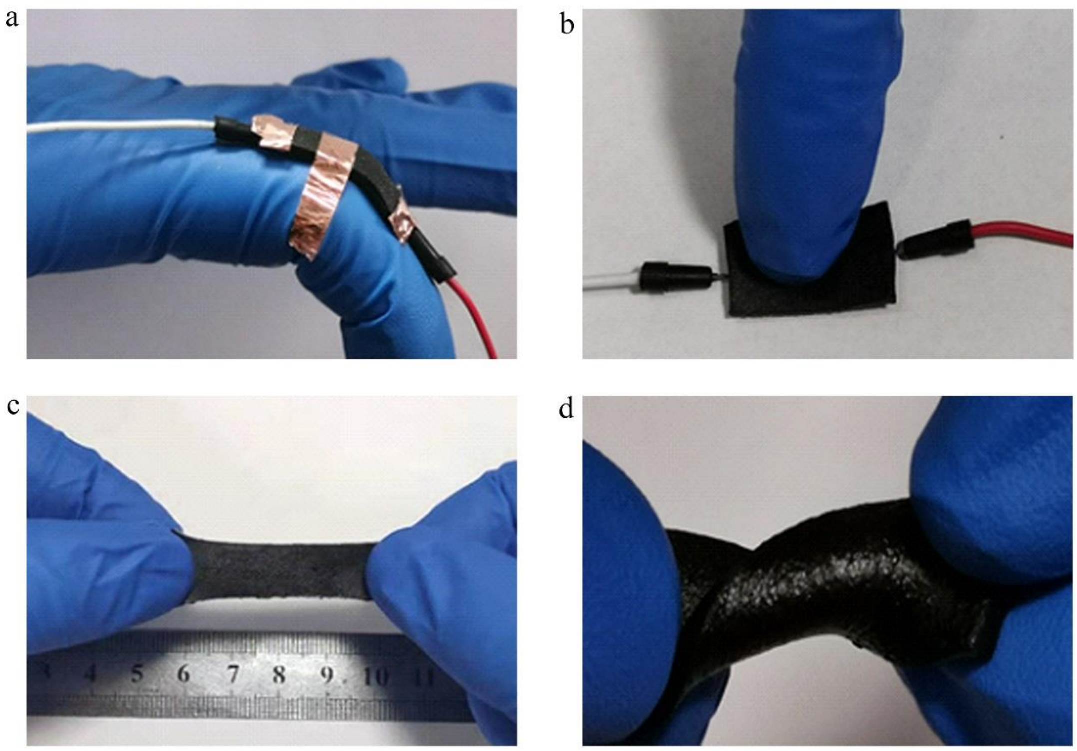

Figure S4. The geometry for the electrodes by different motions.

5. Sensor status for long periods of continuous operation.

When the stimulus is applied for a certain minute at constant pressure, the resistance shows a slight fluctuation, not a constant value. This shows that our sensor is relatively stable.

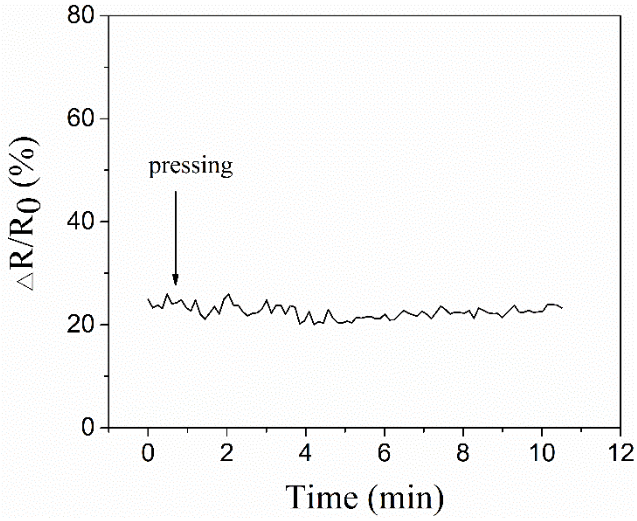

Figure S5. Real-time monitoring of relative resistance changes

6. The preparation of YES logic gate using the temperature responsive conductive hydrogels.

At 40℃, the hydrogel shrinks (input 0) and the circuit is disconnected, so the bulb is not lit (output 0). At 2℃, the hydrogel expands (input 1) and the circuit is switched on, so the bulb lights up (output 1).

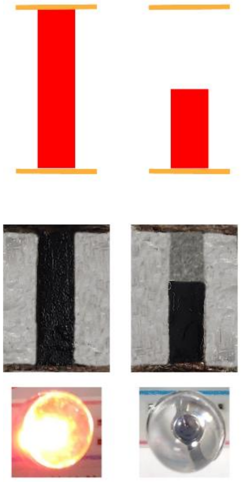

Gel	Output
0	0
1	1

Figure S6. Using temperature responsive conductive hydrogels to prepare YES gate

7. The size of the logic gates

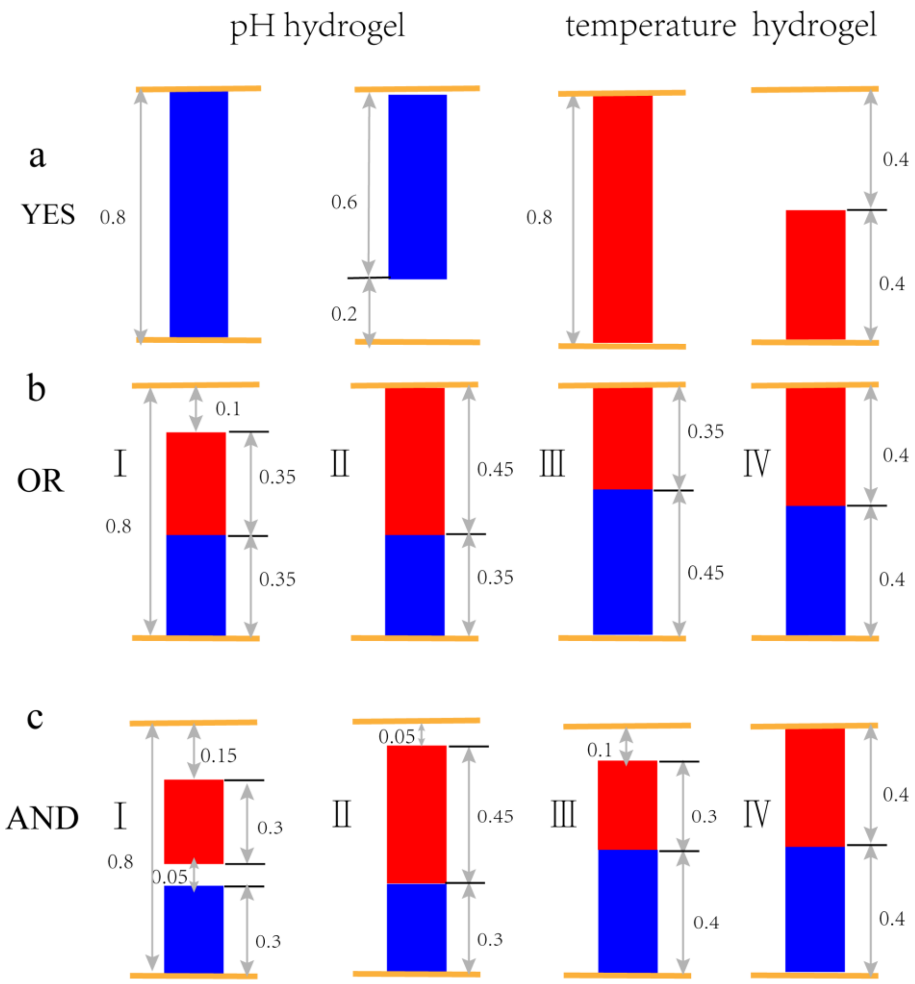

Figure S7. the size of the logic gates(The unit is cm)

8. The swelling ratios of the hydrogels

As shown in the Figure S8 we can find that the pH responsive hydrogel can swell by about 35%, while the temperature sensitive hydrogel can swell by about 50%. Combined with Figure S7 and Figure S8, we can well understand the size of the hydrogel and logic gate when no stimulation is input. When the hydrogel is stimulated, we can contrast the relationship between the dimensions of gap, hydrogel size, logic gate.

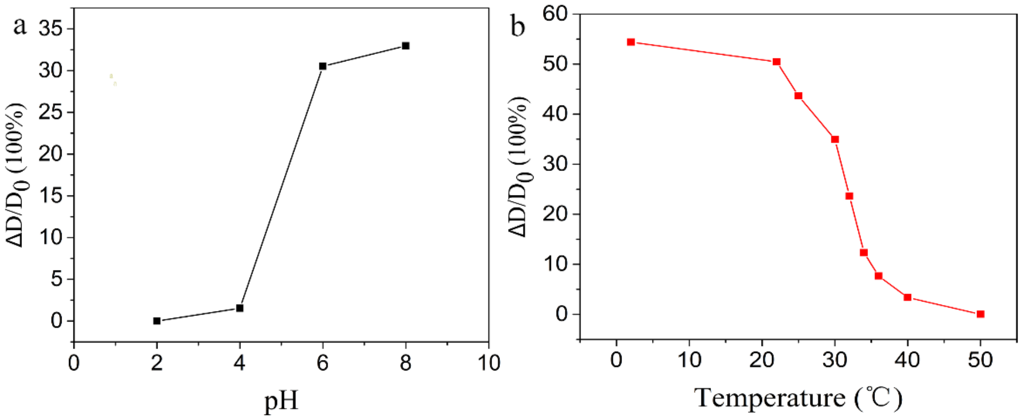

Figure S8. The swelling ratios of the hydrogels. (a) The pH-responsive conductive hydrogel. D_0 represents the diameter of the hydrogel at pH 2, and ΔD represents the difference between the diameter of the other pH and D_0。(b) The thermo-responsive conductive hydrogels. D_0 represents the diameter of the hydrogel at 50℃, and ΔD represents the difference between the diameter of the other temperature and D_0。

Reference

1. Shi, Y.; Ma, C.; Peng, L.; Yu, G. Conductive "smart" hybrid hydrogels with PNIPAM and nanostructured conductive polymers. *Adv. Funct. Mater.* **2015,** *25,* 1219-1225.
2. Sun, N.; Sun, P.; Wu, A.; Qiao, X.; Lu, F.; Zheng, L. Facile fabrication of thermo/redox responsive hydrogels based on a dual crosslinked matrix for a smart on-off switch. *Soft matter* **2018,** *14,* 4327-4334.
